# Nationwide claims data validated for quality assessments in acute myocardial infarction in the Netherlands

**DOI:** 10.1007/s12471-017-1055-3

**Published:** 2017-11-08

**Authors:** D. C. Eindhoven, L. N. van Staveren, J. A. van Erkelens, D. E. Ikkersheim, S. C. Cannegieter, V. A. W. M. Umans, A. Mosterd, J. van Wijngaarden, M. J. Schalij, C. J. W. Borleffs

**Affiliations:** 1 Department of Cardiology, Leiden University Medical Center, Leiden, The Netherlands; 2 Vektis B.V., Zeist, The Netherlands; 3 KPMG-Plexus, Amstelveen, The Netherlands; 4 Department of Epidemiology, Leiden University Medical Center, Leiden, The Netherlands; 5 Department of Cardiology, Noordwest Ziekenhuisgroep, Alkmaar, The Netherlands; 6 Department of Cardiology, Meander Medical Center, Amersfoort, The Netherlands; 7 Department of Cardiology, Deventer Ziekenhuis, Deventer, The Netherlands

**Keywords:** Claims data, Administrative data, Quality of health care, Quality indicator, Acute myocardial infarction, Secondary prevention care

## Abstract

**Introduction:**

Since health insurance is compulsory in the Netherlands, the centrally registered medical claims data might pose a unique opportunity to evaluate quality of (cardiac) care on a national level without additional collection of data. However, validation of these claims data has not yet been assessed.

**Design:**

Retrospective cohort study.

**Methods:**

National claims data (‘national registry’) were compared with data collected by patient records reviews in four representative hospitals (‘validation registry’). In both registries, we extracted the national diagnosis codes for ST-segment elevation myocardial infarction and non-ST-segment elevation myocardial infarction of 2012 and 2013. Additionally, data on medication use at one year after acute myocardial infarction (AMI) was extracted from the Dutch pharmacy information systems and also validated by local patient records reviews. The data were compared at three stages: 1) validation of diagnosis and treatment coding; 2) validation of the hospital where follow-up has taken place; 3) validation of follow-up medical treatment after 365 days.

**Results:**

In total, 3,980 patients (‘national registry’) and 4,014 patients (‘validation registry’) were compared at baseline. After one-year follow-up, 2,776 and 2,701 patients, respectively, were evaluated. Baseline characteristics, diagnosis and individual medication were comparable between the two registries. Of all 52,672 AMI patients in the Netherlands in 2012 and 2013, 81% used aspirin, 76% used P2Y12 inhibitors, 85% used statins, 82% used beta-blockers and 74% angiotensin converting enzyme inhibitors/angiotensin II antagonists. Optimal medical treatment was achieved in 49% of the patients with AMI.

**Conclusion:**

Nationwide routinely collected claims data in patients with an acute myocardial infarction are highly accurate. This offers an opportunity for use in quality assessments of cardiac care.

## Introduction

Acute myocardial infarction (AMI) is the most frequent cause of death in the western world [1]. Numerous studies have shown the beneficial effects of medication following AMI, resulting in increased survival [2–7]. In modern medicine the assessment of quality of care has become increasingly important, especially measurements focussing on patient-relevant outcomes such as mortality and patient experience [8]. Health care professionals have a responsibility to monitor their performances and share these results. However, registration of clinical parameters is laborious and time-consuming, which results in less time for actual patient care [9].

Internationally, claims and clinical databases are explored as a source for quality assessments [10, 11]. The added value of claims databases is the automatic data collection on an ongoing basis, allowing us to follow patients over a longer period of time and cover large populations [12–14]. However, nationwide coverage is scarce. The centrally registered claims data of health care companies in the Netherlands might pose a unique opportunity to evaluate quality of cardiac care on a national level. As all inhabitants of the Netherlands are obliged to have health insurance, almost all inhabitants (99.8%) are registered in a central database (excluding military personnel and prisoners). However, the validity of these claims data has not been assessed.

In the current study, national claims data will be validated for their use in the assessment of quality of care on a national scale. For the validation of claims data, medical treatment in the first year following AMI will be assessed as a first quality indicator.

## Methods

### Validation method

To assess validity of national claims data, four representative hospitals are assessed by comparing claims data of each hospital (‘national registry’) to local data, which is obtained by local patient record reviews (‘validation registry’).

## National registry

In the Netherlands, all reimbursements of the Dutch hospitals are processed by insurance companies and registered by a national diagnosis coding system. These are centrally registered and collected in the Dutch hospital information systems. Furthermore, information on prescribed medication was extracted from the Dutch pharmacy information systems. For the current analysis, we selected all patients admitted in 2012 and 2013 for ST-segment elevation myocardial infarction (STEMI) (coded as 0320.11.204) or non-ST-segment elevation myocardial infarction (NSTEMI) (coded as 0320.11.205). The age, gender and initial treatment (either percutaneous coronary intervention (PCI) or no PCI) of all patients were registered.

The hospital where the first follow-up code was registered was considered the hospital where follow-up had taken place and therefore responsible for medical treatment. If no follow-up code was registered, patients with a PCI were allocated to the centre in which the PCI was performed and patients without a PCI were allocated to the hospital with the first STEMI or NSTEMI code.

For one year following the index event, mortality and the use of the following medication was noted: aspirin (acetylsalicylic acid and carbasalate calcium), P2Y12 inhibitor, statin, beta-blocker, angiotensin converting enzyme (ACE) inhibitor or angiotensin II antagonist (AT2 antagonist). Optimal medical treatment was defined as the usage of these five types of medication. Furthermore, the use of a vitamin K antagonist (VKA) or novel oral anticoagulant (NOAC) was noted as a potential cause for non-usage of aspirin.

### Medication use

Medication use was defined by the registered anatomical therapeutic chemical (ATC) code and the defined daily dose (DDD). The DDD is the assumed average maintenance dose per day for a drug used for its main indication in adults, composed by the World Health Organisation Collaborating Centre for Drug Statistics Methodology [15]. It is a unit of measurement and does not necessarily reflect the recommended or prescribed daily dose. The complete list of the DDD per medication is shown in Table 1. The use of pharmacy records has been validated in previous studies [16].

**Table 1:** Definition of medication use after 365 days

Medication	ATC Code	DDD 365 days
Aspirin	B01AC06 (acetylsalicylic acid) or B01AC08 (carbasalate calcium)	180 DDD
P2Y12 inhibitor	B01AC04 (clopidogrel), B01AC22 (prasugrel) and B01AC24 (ticagrelor)	180 DDD
Statin	C10AAxx (all HMG CoA reductase inhibitors)	180 DDD
Beta-blocker	C07xxxx (all beta-blocking agents and combinations)	45 DDD
ACE inhibitor/AT2 antagonist	C09A, C09B and C09C (including combinations)	120 DDD
Vitamin K antagonist	B01AA04 (phenprocoumon) and B01AA07 (acenocoumarol)	60 DDD
Novel oral anticoagulant	B01AE07(dabigatran etexilate), B01AF01 (rivaroxaban) and B01AF02 (apixaban)	180 DDD

*ACE* angiotensin converting enzyme,* AT2* angiotensin II* ATC* anatomical therapeutic chemical*, DDD* defined daily dose* HMG CoA* hydroxymethyl-glutaryl coenzyme

To increase the algorithm’s sensitivity for detecting medication usage, a threshold of 45 to 180 DDD was used

### National cohort

We assessed baseline data and pharmacological treatment after one-year follow-up of all patients with AMI in the Netherlands in 2012 and 2013.

## Validation registry

**Fig. 1:**
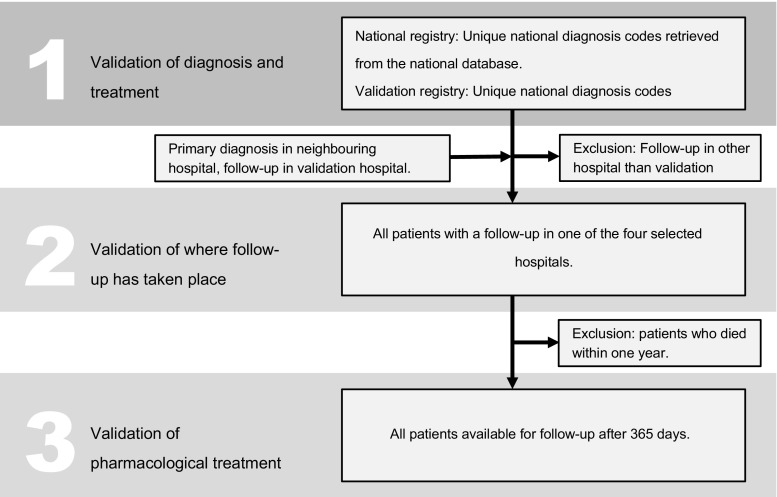
Data from the national registry and validation registry databases are compared at three stages

In the four selected hospitals (an academic PCI centre, two peripheral PCI centres and a non-PCI centre), all variables extracted from the national claims database were collected for validation through patient records reviews. Patients with foreign health insurance were excluded, since these patients were not included in the national analysis. The data from the national registry and validation registry databases were compared at three stages (Fig. 1).

### 1) Validation of diagnosis and treatment coding at first admission

In every hospital, the patient population was selected by using the hospital’s local claims registration database to find all admitted patients with similar national diagnosis codes for STEMI and NSTEMI. In patients with multiple diagnosis codes within the study period, the first admission code was used for the analysis. All codes were checked for the final diagnosis at discharge in which STEMI is defined as ST-elevation in ≥2 contiguous leads with ≥0.1 mV in all leads other than leads V2–V3 and a typical rise and/or fall of cardiac biomarkers [17]. NSTEMI diagnosis is defined by clinical symptoms suggestive for acute ischaemia, with or without ST-segment depression or T‑wave and presence of cardiac biomarkers [18].

### 2) Validation of the hospital where follow-up has taken place

In the validation hospitals, all patient records were screened to assess where follow-up was performed. Patients whose follow-up was performed in another hospital were excluded from follow-up analyses. Additionally, in the non-PCI centre, patients could have been referred from another hospital where the initial AMI was registered, in which case these patients were included for follow-up analysis. Finally, all patients deceased within one year after their first admission were excluded from the follow-up population, similar to the national database, since one-year medication use could not be assessed.

### 3) Validation of pharmacological treatment during one-year follow-up

All remaining patient records were reviewed for medication use.

## Statistical analysis

The data are compared as absolute numbers and as a proportion of the total population. A 95% confidence interval (CI) was calculated with a population proportion formula and indirect comparisons were made by chi-square test between the national registry and validation registry. A *p*-value above 0.05 was considered comparable. Furthermore, a direct comparison of the diagnosis coding in the local database with the final diagnosis at discharge was made.

## Results

### 1)* Validation of diagnosis and treatment*

Of all 59,534 admissions for AMI in the Netherlands in 2012 and 2013, 3,980 (7%) patients were registered in one of the four validation hospitals according to the claim database. Half of the diagnosis codes were for STEMI (51%) (95% CI 49–53%), 68% (95% CI 67–70%) was treated with PCI, mean age was 66 ± 13 years and 68% (95% CI 67–70%) were male.

With patient records reviews in the four hospitals, 4,014 patients were identified. Similar to the national registry, 51% of the patients had a STEMI code (95% CI 49–53%), 69% (95% CI 67–70%) was treated with PCI, mean age was 66 ± 13 and 69% (95% CI 67–70%) were male (Table 2). For the direct validation of the diagnosis codes, the final diagnosis at discharge was correlated with the first diagnosis coding. Due to transfers after treatment, a reliable judgement for final diagnosis was not possible in 254 (6%) patients. Of all remaining 1,977 STEMI codes, 88% was coded correctly as STEMI. Of all 1,738 NSTEMI codes, 93% was coded correctly as NSTEMI.

**Table 2:** Patient characteristics

	National registry	Validation registry	Difference	National cohort
	*N = 3980*	*N = 4014*		*N = 59,534*
Age (yrs)	66 ± 13	66 ± 13		67 ± 13
Male gender	2726 (68%)	2756 (69%)	1%	39,545 (66%)
Deceased <180 days	313 (8%)	292 (7%)	1%	5471 (9%)
Deceased <365 days	388 (10%)	346 (9%)	1%	6862 (12%)
Final diagnosis				
*STEMI*	2028 (51%)	2048 (51%)	0%	25,768 (43%)
*NSTEMI*	1952 (49%)	1966 (49%)	0%	33,766 (57%)
Treated with PCI	2700 (68%)	2754 (69%)	1%	31,632 (53%)

*STEMI* ST-segment elevation myocardial infarction,* NSTEMI* non-ST-segment elevation myocardial infarction, *PCI* percutaneous coronary intervention

### 2) *Validation of the hospital where follow-up has taken place*

To validate the national algorithm of the location where follow-up had taken place, all patients with outpatient follow-up in another centre were excluded. In the national registry, 3,106 patients had their first follow-up code after their AMI was registered in one of the four selected hospitals. Three hundred and thirty patients died within one year and were excluded, resulting in a follow-up population of 2,776 patients.

In the validation registry of 4,014 patients, 1,157 patients received follow-up in another hospital, 146 patients were followed in one of the hospitals although the first admission of AMI was registered elsewhere, and 302 patients died during the first year, resulting in a validated follow-up population of 2,701 patients.

### 3) *Validation of pharmacological treatment*

Analysis of 2,776 patients in the national registry showed aspirin use of 81% (95% CI 79–82%), P2Y12 inhibitor use of 80% (95% CI 78–81%), statin use of 91% (95% CI 90–92%), beta-blocker use of 86% (95% CI 85–87%), ACE inhibitor/AT2 antagonist use of 80% (95% CI 78–81%), VKA use of 17% (95% CI 15–18%) and NOAC use of 1% (95% CI 0–1%) (Table 3).

**Table 3:** Pharmacological treatment during one year follow-up

	National registry	Validation registry	Difference	National cohort
	*N = 2776*	*N = 2701*		*N = 52,672*
*Patients with data available*		*N = 1986*		
Aspirin	2246 (81%)	1689 (85%)	4%*	42,717 (81%)
P2Y12 inhibitor	2207 (80%)	1565 (79%)	1%	39,990 (76%)
Statin	2525 (91%)	1847 (93%)	2%	44,548 (85%)
Beta-blocker	2386 (86%)	1607 (81%)	5%*	43,189 (82%)
ACE inhibitor/AT2 antagonist	2213 (80%)	1656 (83%)	3%	38,795 (74%)
Vitamin K antagonist	465 (17%)	293 (15%)	2%	8669 (16%)
Novel oral anticoagulant	14 (1%)	13 (1%)	0%	184 (0%)
Optimal medical treatment	1665 (60%)	1087 (55%)	5%^*^	25,615 (49%)
*With aspirin*	1439 (52%)	959 (48%)	4%	22,311 (42%)
*With VKA or NOAC*	226 (8%)	128 (6%)	2%	3304 (6%)
At least 4 out of 5	2217 (80%)	1589 (80%)	0%	38,795 (74%)
At least 3 out of 5	2545 (92%)	1863 (94%)	2%	46,885 (89%)

*ACE inhibitor* Angiotensin converting enzyme inhibitor,* AT2 antagonist* angiotensin II antagonist,* VKA* vitamin K antagonist,* NOAC* novel oral anticoagulant

Optimal medical treatment is defined as the use of a combination of aspirin-specie and/or vitamin K antagonist or novel oral anticoagulant, P2Y12 inhibitor, statin, beta-blocker and ACE inhibitor. The replacement of VKA or NOAC is not included in the combined measurements in which patients use at least three or four out of five medications

^*^ *P*-value >0.05 for comparison between the national registry and validation registry

In the validation registry, one-year medication data were available in 1,986 patients, showing comparable rates between the two registries (Table 3).

### National cohort

Of all 59,534 patients (mean age 67 ± 13 years, 66% male), 6,862 (12%) patients died during one-year follow-up (Table 2). Analysis of the remaining 52,672 patients showed aspirin use of 81%, P2Y12 inhibitor use of 76%, statin use of 85%, beta-blocker use of 82%, ACE inhibitor/AT2 antagonist use of 73% (Table 3).

## Discussion

In this study on validation of claims data, the findings can be summarised as follows: (i) the two registries show comparable results for PCI treatment, diagnosis and medical treatment in AMI patients, making it possible to use claims data for quality assessments on a national scale; (ii) medical adherence one year after AMI in the Netherlands is at an acceptable level.

### Claims data and quality assessment

With the growing importance of quality assessment and efficient data collection, routinely collected claims data are being used and studied more frequently for cardiac outcome measurements. Benefits of claims databases are the automatic and continuous data acquisition which make it less laborious for health care professionals to gather health care outcome measurements. Claims data have the advantage of nationwide coverage, depend less on voluntary hospital participation and can be used for chain of care evaluation, including a connection with external datasets. However, claims data are collected predominantly for billing purposes, not for research. Therefore, it requires validation of data accuracy. Data accuracy is particularly important when using claims data as a quality indicator and when comparing medical centres. As previously shown in the United Kingdom, the current study demonstrates a sufficient accuracy of nationwide-used diagnostic, procedural and medical treatment codes with chart review [19]. Other studies on comparisons of claims data with clinical registries show various results [20–22]. This is due to differences in medical specialisation, study purpose and database quality. This analysis composes a large sample size (7% of all Dutch AMI patients) and data quality is verified in various stages. Altogether, the use of these nationwide claims data offer an efficient approach in quality measurement on a national scale. However, claims data contain limited detailed clinical information, which must be taken into account. Adjustment for patient characteristics and case mix is possible up to a certain level, but requires additional evaluation [22, 23].

### Medication compliance following myocardial infarction

Several large trials have shown the beneficial effects of medication on patient outcome [2–7]. Therefore, medication poses a robust indicator for quality of care following AMI [24].

Previous studies focussing on medication at discharge show a substantial guideline adherence in patients with AMI (65–69% were prescribed all five indicated drugs) [25, 26]. Additionally, a recent European guideline evaluation by the EUROASPIRE IV Study Group showed comparable secondary prevention rates at a median follow-up time of 16 months after myocardial infarction: 94% antiplatelets, 86% statins, 83% beta-blockers and 75% ACE inhibitors/AT2 antagonists [27]. Another study from the Netherlands evaluated secondary prevention during long-term follow-up with usage of a similar patient linkage system containing drug-dispensing records from community pharmacies and hospitalised patients and showed an improvement in medication compliance over time (from 1999 till 2007) [28]. When comparing these results with the current study, an additional improvement in medication adherence over time can be observed, suggesting an increased focus on adequate medication prescription after AMI. Approximately half of the currently studied patients do not receive all five indicated drugs one year after AMI. The measured suboptimal adherence of all five indicated drugs is partly physician driven (the drugs are not prescribed, whether or not on purpose) and can be partly patient driven (the patient does not want the medication or is intolerant) [4, 18, 29]. In the guidelines of the European Society of Cardiology, American Heart Association and American College of Cardiology, beta-blocker and ACE inhibitor/AT2 antagonist use is indicated for patients with left ventricular dysfunction (Class 1, level A) and recommended if not contra-indicated in the remaining group [4, 18, 29]. A combined measurement of the five indicated drugs might have its limitations as a quality indicator and therefore requests careful interpretation. Comparison per type of medication poses a more robust indicator for the assessment of quality of care.

### Future implications

National claims data offer a range of possibilities for automated and nationwide quality assessments without additional work for professionals, resulting in more time for actual patient care. Furthermore, national claims data serve as an opportunity in the evaluation of the patient’s chain of care. Future research should focus on the development of additional quality indicators and on the connection between currently available databases, such as primary care databases to assess primary care follow-up, or nursery care databases to assess functional status. In the Netherlands, claims data serve as an additional measurement, alongside the National Heart Registry (NHR) [30]. The NHR could function as a national clinical database with more detailed clinical information. National claims data could be used to check the number in the clinical databases. In the long run, when the field of cardiac professionals is ready, cost effective analysis is possible. A development that can already be seen in the field of surgery, with the Dutch Institute for Clinical Auditing (DICA) and the registration of claims data [31]. All in all, this depends on new laws concerning privacy and the will of politicians, hospitals and clinical professionals.

### Limitations

Some limitations should be addressed. Firstly, differences in adherence between local and national databases may also be caused by different adherence measuring methods; national databases are based on pharmacy fulfilment prescription and local databases are based on self-reporting of the patient, deducted from a chart review. This discrepancy between providing and fulfilment of the prescription is not studied and therefore a small difference between the two registries is in accordance with clinical practice and doesn’t relate to data quality. Secondly, patients were lost to follow-up in the validation registry. Reasons were a physician- or patient-driven conservative approach because of the patient’s terminal disease or high age. Patients who were lost to follow-up may be the patients that used less medication and therefore could probably cause a selection bias of the validation registry.

## Conclusion

Nationwide routinely collected medical claims data in patients with AMI are accurate. This offers an opportunity for use in quality assessments of cardiac care.
